# Trends in Conventional Heart Failure Therapy in a Real-World Multinational ATTR-CA Cohort

**DOI:** 10.3390/jcdd12100403

**Published:** 2025-10-11

**Authors:** Eva H. van der Geest, Nina Ajmone Marsan, Dorien Laenens, Philippe J. M. R. Debonnaire, Mathias Claeys, Fauto Pinto, Dulce Brito, Erwan Donal, Steven Droogmans, Nico Van de Veire, Philippe Bertrand, Takeru Nabeta, Francesca Graziani, Madelien V. Regeer

**Affiliations:** 1 Department of Cardiology, Leiden University Medical Center, 2300 RC Leiden, The Netherlands; 2 Department of Cardiology, Sint-Jan Hospital Bruges, 8000 Bruges, Belgium; 3 Department of Cardiology, AZ Groeninge, 8500 Kortrijk, Belgium; 4 Department of Cardiology, Centro Hospitalar Universitário Lisboa Norte, Faculdade de Medicina, Universidade de Lisboa, 1649-028 Lisboa, Portugal; 5 Cardiologie, CHU de Rennes, Université de Rennes, F-35000 Rennes, France; 6 Centrum voor Hart-en Vaatziekten (CHVZ), Dienst Cardiologie, Universitair Ziekenhuis Brussel (UZ Brussel), Vrije Universiteit Brussel (VUB), 1090 Brussels, Belgium; 7 Department of Cardiology, AZ Maria Middelares, 3600 Gent, Belgium; 8 Department of Cardiology, Hospital Oost-Limburg, 3600 Genk, Belgium; 9 Department of Cardiology, Kitasato University School of Medicine, Sagamihara 8500, Japan; 10 Dipartimento di Scienze Cardiovascolari-CUORE, Fondazione Policlinico Universitario A. Gemelli IRCCS, 00168 Rome, Italy

**Keywords:** transthyretin cardiac amyloidosis, heart failure medication, beta-blockers, angiotensin-converting enzyme inhibitors, angiotensin II receptor blockers

## Abstract

Background: Conventional HF treatment in transthyretin cardiac amyloidosis (ATTR-CA) resulting in restrictive cardiomyopathy is debated due to absent trial evidence in this specific sub-population of heart failure (HF) patients. Current European Society of Cardiology guidelines recommend the use of diuretics and mineralocorticoid receptor antagonists (MRAs). However, beta-blockers (BBs) and angiotensin-converting enzyme inhibitors/angiotensin II receptor blockers (ACEi/ARBs) are often discontinued due to hypotension or bradycardia. This study assesses real-world HF treatment patterns and their impact on survival in a multinational ATTR-CA cohort. Methods: A retrospective analysis of 794 ATTR-CA patients examined baseline BB, ACEi/ARB, and MRA prescriptions. The cohort was divided based on guideline publication dates. Results: Patients were predominantly male (73.2%) with a median age of 78 years. Prescription of diuretics (52.8%) and disease-modifying therapy (44.9%), mostly tafamidis, was common. BBs (43.7%) and ACEi/ARBs (41.2%) were prescribed more often in patients with higher NYHA class, elevated NT-proBNP, and more comorbidities. Blood pressure and heart rate were similar regardless of BB or ACEi/ARB use. BB prescription and combination therapy with BB and ACEi/ARB increased over time. Neither BB nor ACEi/ARB use significantly impacted mortality when analyzed in a multivariate Cox proportional hazard regression. Conclusions: Use of BBs and ACEi/ARBs has increased over time, particularly in advanced-stage ATTR-CA patients, and although these therapies appear to be reasonably tolerated, survival was not significantly altered.

## 1. Introduction

Transthyretin cardiac amyloidosis (ATTR-CA) is characterized by the pathological misfolding of transthyretin proteins, leading to the formation of insoluble amyloid fibrils. These fibrils, originating from either variant (ATTRv) or wild-type (ATTRwt) transthyretin proteins, accumulate in the myocardial interstitium. This deposition disrupts normal cardiac function, manifesting in a spectrum of symptoms such as heart failure (HF), atrial and ventricular arrhythmias, and conduction disorders. The disease predominantly affects elderly males and typically presents as a restrictive cardiomyopathy with preserved ejection fraction and left ventricular hypertrophy [1,2]. In addition, typical electrocardiographic features can be observed in patients with ATTR-CA including low voltages, conduction delay, and pseudonecrosis patterns [3]. The disease can also manifest outside the heart as polyneuropathy, bilateral carpal tunnel syndrome, or lumbar spinal stenosis, which often precedes cardiac involvement.

Significant developments have taken place over the past few years in the diagnosis and treatment of cardiac amyloidosis. A non-invasive diagnostic method has been validated, which uses bone scintigraphy and serum and urine immunofixation as alternatives to cardiac or extracardiac biopsies. This advancement has markedly simplified the diagnostic process and partly led to the discovery that cardiac amyloidosis is more common than previously suspected. Moreover, therapeutic options have greatly improved with the development and introduction of tafamidis, an agent that stabilizes the transthyretin protein, preventing its misfolding and formation of amyloid deposits [4]. More recently, a four-step hierarchical analysis showed that treatment with acoramidis—another TTR stabilizing agent—also led to significantly better outcomes in terms of mortality, morbidity, and function outcomes compared to placebo [5]. In addition, vutrisiran, a subcutaneously administered RNA interference therapeutic agent which inhibits the production of hepatic transthyretin, has been proven beneficial compared to placebo regarding survival and quality of life [6].

Despite advances in diagnostic and therapeutic approaches, the pharmacological management with conventional HF medication in ATTR-CA remains an area of debate. Patients with cardiac amyloidosis are often excluded from the large-scale heart failure trials, resulting in limited evidence on the efficacy and safety of conventional therapies in this population. European Society of Cardiology (ESC) guidelines on the diagnosis and management of acute and chronic heart failure have changed over time and make a distinction in treatment of patients with heart failure with reduced ejection fraction (HFrEF) and patients with heart failure with preserved ejection fraction (HFpEF). In all heart failure patients, fluid overload should be managed using loop diuretics The recommendations from 2016 advise to treat all HFrEF patients with beta-blockers, angiotensin-converting enzyme inhibitors (ACEi) or angiotensin receptor blockers (ARBs), and mineralocorticoid receptor antagonist (MRA) when left ventricular ejection fraction (LVEF) falls below 35%. For HFpEF and ATTR-CA patients, no specific recommendations were made [7]. In 2021, both the updated ESC guidelines on heart failure and the ESC position statement on the diagnosis and treatment of cardiac amyloidosis (CA) were published [8,9]. In these new guidelines, there is a central role for sodium-glucose transport protein 2 (SGLT2) inhibitors in all heart failure patients regardless of the LVEF. Furthermore, patients with an LVEF between 40% and 50% may be treated as HFrEF patients. Specifically for CA patients, there could be a role for MRAs. However, the use of other standard heart failure therapies, such as beta-blockers, ACEi, or ARBs, is frequently limited because of intolerance due to orthostatic hypotension. Therefore, their value in the treatment of ATTR-CA patients remains unclear [8,9,10].

To explore what prescription patterns occur in daily practice, a study was performed within the National Amyloidosis Centre in London [11]. Ioannou et al. describe that a relatively small proportion of their ATTR-CA cohort was treated with conventional heart failure medication. This proportion was more likely to have a more severe HF phenotype. Beta-blockers and ACEi/ARBs were frequently prescribed in low doses or discontinued. Only in patients with LVEF <40% was the use of beta-blockers associated with a small survival benefit in a propensity score-matched cohort. This London study was conducted among a patient cohort treated at a highly specialized amyloidosis center, which is potentially less representative for real-world all-comer ATTR populations. Although ATTR-CA is more prevalent than previously appreciated, it remains a rare condition, and usually the number of amyloidosis patients under care in secondary and tertiary centers is small. This raised the question what patterns of beta-blocker and ACEi/ARB prescription occur in these types of centers.

The aim of this study is to evaluate, in a real-world multinational ATTR-CA patient cohort, (1) the prescription patterns of conventional heart failure therapy in ATTR-CA patients, (2) what changes have occurred in these prescriptions patterns as guidelines have been revised, and (3) whether the type of HF therapy is associated with all-cause mortality.

## 2. Materials and Methods

The primary analysis was performed on a retrospective multinational cohort of patients diagnosed with cardiac amyloidosis from 19 centers (Argentina, Australia, Belgium (=5), Brazil, France, Hong Kong, Israel, Italy, Japan (=3), Portugal, Romania, Singapore, and the Netherlands). Patients diagnosed between 1996 and 2024 were included. Only patients with ATTR-CA, after light chain (AL) amyloidosis was ruled out, were selected for further analysis. Exclusion criteria included incomplete typing of amyloidosis and absence of cardiac manifestation of amyloidosis. The institutional review board approved this retrospective analysis of clinically acquired data and waived the need for patient written informed consent.

The data recorded in the database consisted of medical history/comorbidities, baseline medications, laboratory data, electrocardiogram (ECG) measurements, and results of diagnostic imaging (bone scintigraphy and cardiac magnetic resonance). Outcome data, i.e., death, HF hospitalization, stroke, new onset of atrial fibrillation (AF) or atrial flutter (AFL), and pacemaker/implantable cardioverter-defibrillator (ICD) implantation, were recorded. Baseline conventional heart failure treatment with the following medication was recorded: beta-blockers, ACEi/ARBs, MRAs, and loop diuretics.

For the description of baseline characteristics, the patient cohort was split based on LVEF. The most recent ESC guidelines [9] divide heart failure into three groups: heart failure with reduced ejection fraction (HFrEF, LVEF ≤ 40%), mildly reduced ejection fraction (HFmrEF, LVEF 40–50%), and preserved ejection fraction (HFpEF, LVEF ≥ 50%). However, previous guidelines distinguish only HFrEF from HFpEF. Because the characteristics of HFmrEF are most similar to those of HFrEF, it was decided to merge them for analysis and adopt an LVEF of 50% as a cut-off value.

In order to classify patients based on their disease progression, the NAC score was calculated. This score, developed in 2018 by Gillmore et al., stratifies patients with ATTR-CA into prognostic categories based on NT-proBNP levels and eGFR. Levels of NT-proBNP ≤ 3000 ng/L and eGFR ≥ 45 mL/min are classified as Stage I, NT-proBNP > 3000 ng/L and eGFR < 45 mL/min are defined as Stage III, and the residual cases fall within Stage II [12]. ATTR-CA patients typically present with apical sparing on LV longitudinal strain assessment, resulting in the so-called “cherry on top” pattern. The relative apical sparing ratio (RELAS) of longitudinal strain was calculated in order to quantify this phenomenon [13].

**Figure 1:**
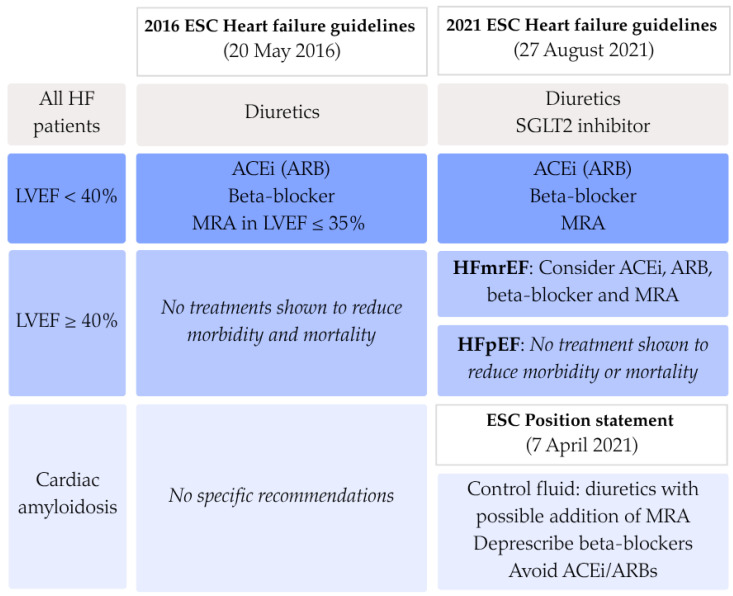
Overview of European guidelines published on the pharmacological treatment of heart failure in cardiac amyloidosis. ACEi: angiotensin converting enzyme inhibitor; ARB: angiotensin receptor blocker; ESC: European Society of Cardiology; HFmrEF: heart failure with mildly reduced ejection fraction; HFpEF: heart failure with preserved ejection fraction; MRA: mineralocorticoid receptor antagonist; SGLT2: sodium-glucose transport protein 2.

For the analysis of the prescription patterns over time, patients were split into three groups based on the date of their ATTR-CA diagnosis. Dates of official guideline publications were used as cut-off points. The first group was defined as having been diagnosed before the publication of the 2016 ESC Guidelines for the diagnosis and treatment of acute and chronic heart failure, i.e., 20 May 2016. The second group extends until 7 April 2021, when the Diagnosis and treatment of cardiac amyloidosis: a position statement of the ESC Working Group on Myocardial and Pericardial Diseases was published. Later that same year, the ESC Heart Failure guidelines were updated but without significant changes regarding conventional heart failure treatment in CA patients in comparison to the ESC position statement. Therefore, the last group was defined as every patient diagnosed after 7 April 2021 until data cut-off of 29 January 2025. An overview of the recommendations made in the three published documents is shown in Figure 1.

Categorical variables are shown as frequencies and percentages. Continuous variables are presented as mean ± standard deviation (SD) or median with interquartile range [IQR], depending on whether the data were normally distributed, which was assessed graphically. To compare continuous variables between two groups, Student’s *t*-test or the Mann–Whitney U test was used, depending on whether or not the variable followed a normal distribution. NT-proBNP values were log-transformed for bivariate testing. Analysis of Variance (ANOVA) or the Kruskal–Wallis test were used for comparing continuous data across more than two groups. Categorical data was compared using the χ^2^ test. A *p*-value of <0.05 was considered statistically significant. To compare the proportions of HF treatment use over different years, a logistic regression was used, displaying odds ratios with their 95% confidence intervals as OR (95% CI).

The mortality endpoint was defined as time from date of diagnosis to death for all deceased patients or date of diagnosis to date of censoring for all remaining patients. Follow-up was restricted to 48 months. Kaplan–Meier survival analysis was applied, and the log-rank test was employed to evaluate the statistical significance of differences in survival between groups with and without HF medication prescription. For multivariate Cox regression analysis, baseline variables were tested in a univariate Cox model. Significant variables were thereafter added to the multivariate Cox regression model to analyze survival by type of heart failure medication, with the restriction that there were required to be at least 10 events per included variable. Hazard ratios (HRs) with 95% confidence intervals were provided.

All statistical analyses were performed in IBM SPSS Statistics (version 29). Survival curves were generated in GraphPad Prism (version 10.2.3).

## 3. Results

### 3.1. Prescription Patterns

An overall ATTR-CA population of 794 patients was identified, of whom 73.2% were male, and the median age was 78 years. In 680 patients, genetic testing was performed, with 30.6% diagnosed with hereditary ATTR and 69.4% with the wild-type form. Among patients with ATTRv, a Val30Met mutation was the most common genotype (62.0%), followed by a Glu54Gln mutation (9.9%). Approximately half (46.7%) of the patients had a history of AF or atrial flutter at baseline. The majority fell in NYHA classes I and II (78.0%) and NAC stage 1 (59.6%). The median N-terminal pro-B-type natriuretic peptide (NT-proBNP) and high-sensitivity (HS) troponin levels exceeded the upper limit of normal, reaching 1508 ng/L and 48 ng/L on average, respectively. Patients often suffered from chronic kidney disease stage 3 or worse, and their mean estimated glomerular filtration rate (eGFR) was 65.0 mL/min/1.73 m^2^. Echocardiography showed a mean LVEF of 52.1%, left ventricular hypertrophy and diastolic dysfunction with high mitral E/e’ ratio, and increased left atrial volume. Of the conventional heart failure medications, diuretics were most often prescribed (52.8%). Beta-blockers and ACEi/ARBs were prescribed in 43.7% and 41.2% of patients, respectively. In a quarter of patients (25.8%), an MRA was prescribed. Only in the 90 most recently diagnosed patients was the prescription of SLGT2 inhibitors documented. Of them, 18.9% were prescribed an SGLT2 inhibitor. During follow-up, 44.9% of patients used disease-modifying treatment, of whom 89.3% used tafamidis.

Based on LVEF, patients with HF(m)rEF, i.e., LVEF < 50% (*n* = 249, 38.5%), were compared to patients with HFpEF, i.e., LVEF ≥ 50% (*n* = 397, 61.9%). Patients with an LVEF < 50% were more often male, were older, and more often had a history of AF/flutter (62.8% vs. 40.5%, *p* < 0.001). Their functional status based on NYHA class was worse. Additionally, the disease had often progressed further, with approximately one-third of patients in each NAC stage, compared to two-thirds of patients with a preserved LV ejection fraction being in NAC stage 1. Patients presented with higher NT-proBNP (1782 vs. 1212 ng/L, *p* < 0.001) and higher HS troponin levels (57 vs. 40 ng/L, *p* < 0.001). Kidney function based on eGFR was worse (55.6 vs. 67.9 mL/min/1.73 m^2^, *p* < 0.001) and systolic blood pressure was lower. Echocardiographic measurements showed an overall significantly more progressed cardiac disease, with worse global left ventricle longitudinal strain (LV GLS). Relative apical sparing, a typical feature of CA, was similar across both groups, with mean RELAS of 2.0 and 2.1. Proportionally, more patients were prescribed beta-blockers (56.0% vs. 40.8%, *p* < 0.001), diuretics (72.9% vs. 44.2%, *p* < 0.001), and MRAs (35.5% vs. 22.0%, *p* < 0.001). ACEi/ARB use was similar across both groups. All baseline characteristics are displayed in Table 1. Baseline characteristics of patients with beta-blocker and ACEi/ARB prescription are separately displayed in Table 2.

#### 3.1.1. Beta-Blockers

Patients who were prescribed a beta-blocker at baseline were older and more frequently had a history of AF/flutter (60.3 vs. 36.8%, *p* < 0.001). Comorbidities such as coronary artery disease (CAD), diabetes mellitus (DM), hypertension, and stroke or transient ischemic attack (TIA) were more prevalent in patients treated with beta-blockers. The patients presented with more advanced NYHA and NAC stages and had higher NT-proBNP and HS troponin levels. Renal function was significantly lower, and a larger percentage was classified to have chronic kidney disease stage 3 or worse. Blood pressure and heart rate were not significantly different between the beta-blocker and non-beta-blocker groups. Patients with beta-blocker prescription had a lower mean LVEF (50.0 vs. 53.8%. *p* < 0.001) and exhibited signs of more advanced cardiac remodeling and impaired cardiac function on echocardiography, including a thicker interventricular septum, reduced LV GLS, and impaired right ventricle systolic function. Increased left atrial volume and elevated filling pressures indicated that patients treated with beta-blockers had a worse diastolic cardiac function. They were more often prescribed a diuretic or MRA than patients without beta-blocker prescription. On the other hand, the prescription of disease-modifying therapies was less frequent in beta-blocker users (38.3 vs. 49.9%, *p* = 0.002).

#### 3.1.2. Angiotensin-Converting Enzyme Inhibitors/Angiotensin Receptor Blockers

Patients with a ACEi/ARB prescription were more often male, had a higher age at diagnosis, and more frequently had a history of AF/flutter, CAD, and stroke or TIA. More patients had hypertension (71.7% vs. 39.3%, *p* < 0.001); however, systolic or diastolic blood pressure were not significantly different from the non-ACEi/ARB group at baseline evaluation. They presented with a more advanced NAC stage and with higher NT-proBNP levels (2796 vs. 2285 ng/L, *p* < 0.001). Renal function, based on eGFR, was lower in the patients with ACEi/ARB use, with eGFR 60.8 vs. 68.6 mL/min (*p* < 0.001) and 50.7 vs. 38.0% of patients classified in CKD stage of 3 or higher (*p* < 0.001). On echocardiography, in patients treated with ACEi/ARBs, thicker interventricular septum, larger left atrium, and reduced LV GLS were measured. Patients who were treated with ACEi/ARBs were more often prescribed a diuretic or MRA. The proportion treated with disease-modifying treatment was not significantly different.

### 3.2. Trends in Prescription Patterns

#### 3.2.1. Beta-Blocker and ACEi/ARB Prescription

**Figure 2:**
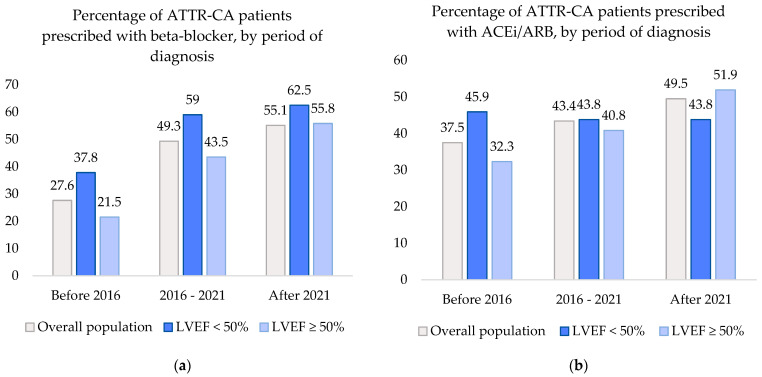
Percentages of patients in the ATTR-CA cohort prescribed with beta-blockers and ACEi/ARBs, by period of diagnosis. (**a**) Beta-blocker prescription percentages in the overall ATTR-CA population and split by LVEF, by period of diagnosis. (**b**) ACEi/ARB prescription percentages in the overall ATTR-CA population and split by LVEF, by period of diagnosis. ATTR-CA: transthyretin cardiac amyloidosis; ACEi/ARB: angiotensin-converting enzyme inhibitor/angiotensin receptor blocker; LVEF: left ventricular ejection fraction.

In the overall population, prescription of beta-blockers and ACEi/ARBs has increased over the past decade. The proportion of patients who were treated with beta-blockers and ACEi/ARBs in each of the three time periods (before 2016, 2016–2021, and after 2021) is graphically displayed in Figure 2.

The percentage of beta-blockers prescribed in ATTR-CA patients showed a significant increase of 27.% before 2016 to 55.1% after 2021 (*p* < 0.001). Compared to before 2016, the odds of beta-blocker use were 2.5 times higher in 2016–2021 (95% CI 1.7–3.8) and 3.2 times higher after 2021 (95% CI 1.9–5.4). ACEi/ARB prescription also displayed an increase from 37.5% to 49.5%. However, this increase was not found to be significant with OR 1.3 (95% CI 0.9–1.9) between 2016 and 2021 and OR 1.6 (95% CI 1.0–2.7) after 2021 (*p* = 0.169).

When dividing the cohort into patients with LVEF < 50% or LVEF ≥ 50%, the changes in heart failure medication prescription remained consistent with those observed in the overall cohort; i.e., a significant increase was seen in beta-blocker use and a non-significant change in ACEi/ARB use. In patients with LVEF < 50%, the odds of beta-blocker prescription increased with ORs of 2.4 (95% CI 1.1–4.9) and 2.7 (95% CI 1.0–7.0) (*p* = 0.046) and ACEi/ARB prescription showed ORs of 0.9 (95% CI 0.5–1.9) and 0.9 (0.4–2.4) (*p* = 0.971). In patients with LVEF ≥ 50%, beta-blocker use increased with OR 2.8 (1.5–5.3) and 4.6 (2.1–10.3) (*p* < 0.001). ACEi/ARB use increased with OR 1.4 (0.8–2.6) and 2.3 (1.1–4.8) (*p* = 0.100).

#### 3.2.2. Combination Heart Failure Therapy

In general, the majority (69.7%) of the ATTR-CA patient population was prescribed some type of conventional HF medication, which refers to beta-blockers, ACEi/ARBs, and MRAs. A combination therapy of these three agents was frequently prescribed. In the overall ATTR-CA cohort, the combination of beta-blocker and ACEi/ARB was the most common, with 23.1% of patients receiving both medications, as part of dual therapy or triple therapy. Of the three agents, MRAs were prescribed least frequently. In case an MRA was prescribed, it was most often as part of combination therapy. Therapy with ACEi/ARB and MRA together was the least common form of combination therapy. Frequencies of each combination of the three types of heart failure medication are displayed in Table 3.

**Figure 3:**
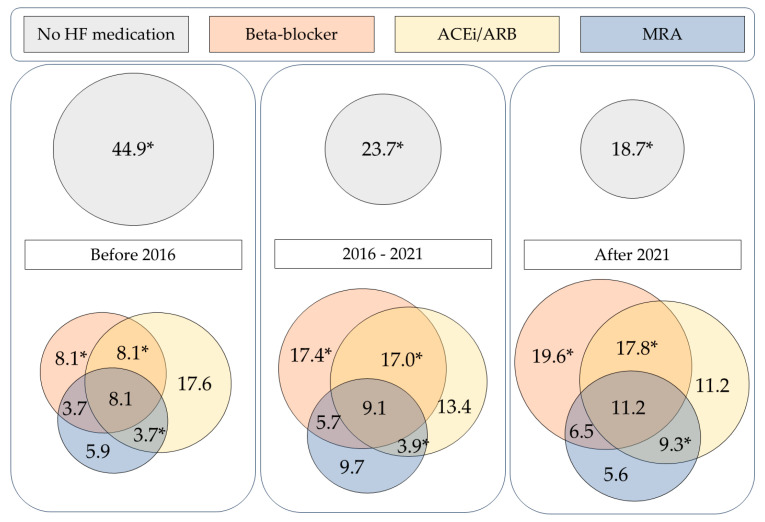
Combination heart failure therapy of beta-blockers, ACEi/ARBs, and MRAs in the overall ATTR-CA population and split by date of ATTR-CA diagnosis (% of population). * *p* < 0.05. ACEi/ARB: angiotensin-converting enzyme inhibitor/angiotensin receptor blocker; HF: heart failure; MRA: mineralocorticoid receptor antagonist.

The prescription of combination heart failure therapy has changed over time. The combination of beta-blockers and ACEi/ARBs was used more frequently in recent years, increasing from 8.1% before 2016 to 17.8% after 2021 (*p* = 0.030). A similar trend was observed for the combination of ACEi/ARB with MRA, which rose from 3.7% to 9.3% over the same period (*p* = 0.042). Meanwhile, the proportion of patients who received none of these three heart failure drug classes (BB, ACEi/ARB, or MRA) decreased significantly, from 44.9% to 18.7% (*p* < 0.001). Figure 3 visually depicts the use of combinations of heart failure therapy, across the cohort and by period of diagnosis.

### 3.3. Heart Failure Medication and Survival

**Figure 4:**
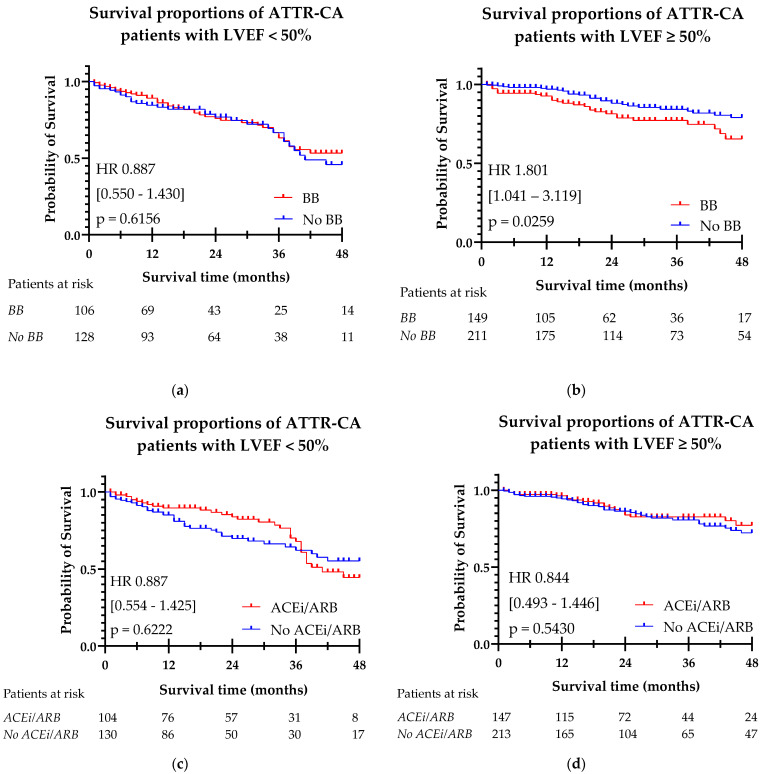
Kaplan–Meier curves of beta-blocker (**a**,**b**) and ACEi/ARB (**c**,**d**) prescription in ATTR-CA patients, split by LVEF < 50% and LVEF ≥ 50%. (**a**) Survival in patients treated with beta-blockers in ATTR-CA patients with LVEF < 50%. (**b**) Survival in patients treated with beta-blockers in ATTR-CA patients with LVEF ≥ 50%. (**c**) Survival in patients treated with ACEi/ARBs in ATTR-CA patients with LVEF < 50%. (**d**) Survival in patients treated with ACEi/ARBs in ATTR-CA patients with LVEF ≥ 50%. ACEi/ARB: angiotensin-converting enzyme inhibitor/ angiotensin receptor blocker; ATTR-CA: transthyretin cardiac amyloidosis; BB: beta-blocker; LVEF: left ventricle ejection fraction.

The Kaplan–Meier curves for beta-blocker and ACEi/ARB use with hazard ratios, confidence intervals, and significance levels are shown in Figure 4. In a Kaplan–Meier survival analysis, beta-blocker use was associated with a higher risk of mortality in patients with an LVEF ≥ 50% (HR 1.801 (95% CI 1.041–3.119), *p* = 0.0259). In patients with LVEF < 50%, no significant difference in survival was observed. ACEi/ARB prescription was not found to be associated with a significant risk change in mortality in both LVEF < 50% and LVEF ≥ 50%. Additionally, survival differences between beta-blocker and ACEi/ARB use in relation to ATTR-CA phenotype (wild-type vs. variant) were analyzed. No significant survival differences were found between these groups. The Kaplan–Meier curves illustrating these comparisons are provided in the Supplementary Materials in Figure S1.

Considering the significant differences found in the baseline populations of beta-blocker and ACEi/ARB users, a multivariable Cox regression analysis was performed. Seven covariables (age, AF, CAD, DM, wild-type phenotype, NAC stage, and LV GLS) were added to the model in addition to beta-blocker or ACEi/ARB use, as depicted in Table 4. The results of univariate testing of these variables are presented in the Supplementary Materials in Table S1. After the model was corrected for these covariates, no significant difference in mortality was found for patients who were prescribed a beta-blocker compared to patients without beta-blocker (HR 0.67 (95% CI 0.352–1.262), *p* = 0.213). ACEi/ARB prescription remained not to show significant change in mortality (HR 0.88 (95% CI 0.49–1.58), *p* = 0.663). When the cohort was split into LVEF < or ≥ 50%, no significant difference in survival was found between patients with or without beta-blocker or ACEi/ARB.

## 4. Discussion

This study demonstrates that the majority of patients with ATTR-CA receive conventional heart failure therapies. Limited research has been conducted on the use of HF medication in this specific patient group, which complicates putting the numbers found in perspective. However, some data are available regarding their prescription in the broader heart failure population, not specifically limited to patients with amyloidosis-related heart failure. For example, the 2024 TITRATE-HF study provided insights on the implementation of heart failure guidelines in a large cohort of heart failure patients in the Netherlands, revealing a high rate of guideline-directed medical therapy (GDMT) use within this overall heart failure population [14]. Specifically, approximately 80% of patients were prescribed ACEi, beta-blockers, and MRAs in accordance with established guidelines, although often at suboptimal doses. Notably, about half of the patients had already been using one more conventional HF agents for other conditions prior to being diagnosed with heart failure.

In the study by Ioannou et al., HF medication prescription was examined in a large ATTR-CA cohort treated in the National Amyloidosis Centre in London [11]. The reported medication use in this cohort was lower: 55% of patients were prescribed beta-blockers, 57% ACEi/ARBs, and 39% MRAs. Although Ioannou et al. did not explicitly assess whether medication use adhered to guideline recommendations, the general discouragement of beta-blockers and ACEi/ARBs in ATTR-CA suggests that the proportion of patients receiving HF medications according to guidelines is considerably lower compared to the broader HF cohort. Moreover, similar to the TITRATE-HF study, a substantial proportion of patients received suboptimal doses of beta-blockers or ACEi/ARBs.

Our study corroborates these findings, indicating that conventional HF therapies are still quite commonly prescribed in ATTR-CA and are generally used in more advanced clinical stages of the disease. Although beta-blockers and ACEi/ARBs have historically been and continue to be the most frequently prescribed HF medications, they remain the most commonly discouraged agents for ATTR-CA because of hypotension and intolerability.

Beta-blockers are commonly indicated for conditions such as coronary artery disease and atrial fibrillation. However, it is noteworthy that in our study, beta-blockers were prescribed to an increasing number of patients without a history of atrial fibrillation, suggesting a broader use of these agents in the management of heart failure in ATTR-CA. A similar pattern was observed for ACEi/ARBs, which are typically prescribed for hypertension. The findings of this study revealed no significant differences in baseline blood pressures between patients using ACEi/ARBs and those not using them, despite the higher prevalence of hypertension among the former. This suggests that ACEi/ARBs are prescribed selectively to patients who are likely to benefit from their effects and who can tolerate the treatment, although this study did not specifically examine adverse events.

Comparing baseline characteristics between groups based on whether or not they are prescribed medication remains a challenge. Establishing clear associations is difficult and based on this data it cannot be determined which came first: the worse clinical condition or the medication use. Despite this, one important observation is that patients using beta-blockers did not exhibit significantly lower heart rates or blood pressure at baseline evaluation. Interestingly, in our cohort, no significant differences in blood pressure or heart rate were observed between patients receiving beta-blockers or ACEi/ARBs and those who were not. This suggests that these patients who were prescribed ACEi/ARBs and beta-blockers have tolerated these agents without experiencing common adverse effects, such as bradycardia or hypotension, which are often cited as reasons for discontinuation [15]. This contrasts with the results from the London study, where both beta-blocker and ACEi/ARB users had lower systolic and diastolic blood pressures, and beta-blocker users demonstrated a lower mean heart rate. This discrepancy raises questions about the generalizability of the assumption that patients with ATTR-CA who are prescribed conventional HF therapies tolerate them well. Further investigation is needed to determine whether these medications are tolerated at which dosages in this patient population.

Alternatively, in HFrEF patients, angiotensin receptor/neprilysin inhibitor (ARNI) can be prescribed instead of ACEi/ARB. Limited evidence of ARNI in six elderly ATTR-CA patients shows that it might be tolerated at low dosage [16]. However in general, ARNIs are associated with hypotension, in particular in HFpEF and HFmrEF patients, and are therefore probably less tolerated in ATTR-CA patients [17].

MRAs were most frequently prescribed as part of combination therapy, typically alongside beta-blockers and/or ACEi/ARBs. MRA monotherapy was rarely used in ATTR-CA patients, which suggests that MRAs are more often added as a second or third choice therapy for heart failure. This observation is in contrast to the ESC heart failure guidelines, which recommend the use of loop diuretics with the potential addition of an MRA as an initial treatment strategy for cardiac amyloidosis. In this study, the focus was placed on beta-blockers and ACEi/ARBs, as these agents are most prominently discouraged in the guidelines. However, further research is needed to better understand the rationale behind MRA prescriptions and to investigate the reasons for their underuse.

The use of SGLT2 inhibitors in clinical practice is a notable emerging trend. Early clinical trials have shown promising results in the ATTR-CA population [18,19]. However, while SGLT2 inhibitors have demonstrated potential therapeutic benefits, further research involving larger patient cohorts is needed to establish clear prescription patterns and clinical outcomes in ATTR-CA patients. In this study, the prescription of SGLT2 inhibitors at the time of diagnosis was not yet common practice and therefore no formal statistical analysis was performed on the use of SGLT2 inhibitors.

The evidence linking beta-blocker use to mortality and adverse outcomes in ATTR-CA patients remains inconclusive. Some studies have failed to establish a direct association between beta-blocker therapy and mortality outcomes [20], while others suggest that beta-blockers may reduce all-cause mortality [21,22]. In our multivariate analysis, corrected for age, AF, CAD, DM, hypertension, NAC stage, and GLS, there was no significant difference in survival with or without beta-blocker use. A small Italian study found that beta-blocker therapy was especially poorly tolerated in patients with AL rather than ATTR amyloidosis and that the intolerance to beta-blockers was associated with hemodynamic function [23]. Additionally, patients on ACEi/ARBs did not experience a higher incidence of adverse events compared to other groups. Instead of HF medication use, lower stroke volume and cardiac output were found to be better predictors of heart failure-related hospitalizations. Lower LVEF was associated with increased risk of hypotension. Furthermore, this Italian study showed that discontinuation of beta-blocker therapy significantly reduced the risk of mortality, although the clinical implications of these findings remain uncertain. On the contrary, the study by Ioannou et al. found that low-dose beta-blocker use was independently associated with a reduced risk of mortality in ATTR-CA patients with reduced ejection fraction [11]. This finding underscores the need for further research into the impact of beta-blocker dosage on survival outcomes in this population.

### Limitations

This study is limited by its retrospective nature and lack of randomization or propensity score matching. There was no information on dosage and longitudinal changes in the prescribed medication during follow-up. Therefore, more in-depth analysis on the effect of dosing on mortality could not be performed. The decision to discourage or promote the use of beta-blockers and ACEi/ARBs cannot be made based on these results. Therefore, more research on the use of heart failure medications and their impact on survival in the cardiac amyloidosis population is necessary, while considering dosing, subtyping of amyloidosis, and clinical disease stage and including SGLT2 inhibitors in a larger study population. This study reflects real-world practice in non-expert secondary and tertiary centers in care for cardiac amyloidosis. Only half of the patients were treated with disease-modifying drugs (most often Tafamidis), because of local regulations and availability. As more patients will be diagnosed at an earlier stage and qualify for specific disease-modifying treatment, the spectrum of CA patients will probably shift to more asymptomatic patients with preserved ejection fraction. It remains uncertain whether there is benefit of beta-blockers and ACEi/ARBs in these patients. The conflicting evidence on the relationship between HF therapy use, intolerance, and mortality in ATTR-CA highlights the need for further research to identify subgroups of patients who may benefit from these therapies. Future studies should focus on both early- and late-stage patients to provide the best supportive care with conventional heart failure treatment next to the more expensive disease-modifying drugs.

## 5. Conclusions

Beta-blockers and ACEi/ARBs are still frequently prescribed in ATTR-CA patients, despite being discouraged in guidelines. Patients who receive beta-blockers or ACEi/ARBs tend to have an overall worse clinical condition. Conventional HF medication use and combination HF therapy have increased over the past decade. When corrected for comorbidities, beta-blocker or ACEi/ARB prescription does not seem to have an association with mortality.

## Figures and Tables

**Table 1:** Baseline characteristics, echocardiographic parameters, and heart failure medication use of overall ATTR-CA population and split by LVEF < 50% and LVEF ≥ 50%.

	Overall Population (*n* = 794)	LVEF < 50% (*n* = 249, 38.5%)	LVEF ≥ 50% (*n* = 397, 61.5%)	*p*-Value
Sex (male)	581 (73.2%)	200 (80.3%)	290 (73.0%)	0.036
Age	78 (66–83)	80 (73–84)	78 (64–83)	0.001
AF/flutter in history	329 (46.7%)	142 (62.8%)	137 (40.5%)	<0.001
CAD	178 (22.4%)	68 (27.3%)	87 (22.0%)	0.122
DM	121 (15.3%)	49 (19.8%)	52 (13.2%)	0.025
Hypertension	417 (52.8%)	128 (51.8%)	224 (56.7%)	0.206
Stroke/TIA	87 (13.4%)	35 (17.7%)	41 (13.1%)	0.157
NYHA class				<0.001
I	217 (30.6%)	36 (14.9%)	120 (33.6%)	
II	336 (47.4%)	132 (54.8%)	174 (48.7%)	
III	138 (19.5%)	66 (27.4%)	53 (14.8%)	
IV	18 (2.5)	7 (2.9%)	10 (2.8%)	
NAC stage				<0.001
I	319 (59.6%)	55 (34.8%)	189 (65.9%)	
II	124 (23.2%)	54 (34.2%)	60 (20.9%)	
III	92 (17.2%)	49 (31.0%)	38 (13.2%)	
NT-proBNP (ng/L)	1508 (347–4009)	1782 (3650–6918)	1212 (312–3265)	<0.001
HS Troponin (ng/L)	48 (28–82)	57 (41–100)	40 (21–68)	<0.001
eGFR (mL/min/1.73 m^2^)	65.0 ± 25.1	55.6 ± 20.8	67.9 ± 24.0	<0.001
CKD stage 3–5	319 (43.3%)	135 (57.7%)	147 (38.5%)	<0.001
Systolic blood pressure (mmHg)	127.5 ± 21.4	124.6 ± 21.8	129.2 ± 21.0	0.012
Diastolic blood pressure (mmHg)	73.8 ± 13.2	74.0 ± 14.3	73.5 ± 12.6	0.640
Electrocardiography				
Rhythm at baseline				0.003
Sinus rhythm	470 (59.9%)	124 (50.2%)	253 (64.9%)	
AF/flutter	248 (31.6%)	100 (40.5%)	114 (29.2%)	
Paced rhythm	62 (7.9%)	22 (8.9%)	21 (5.4%)	
Heart rate (bpm)	74.7 ± 15.9	76.1 ± 18.3	73.1 ± 13.9	0.028
Conduction abnormalities				<0.001
LBBB	125 (18.0%)	56 (26.0%)	59 (16.6%)	
RBBB	94 (13.5%)	32 (14.9%)	49 (13.8%)	
Aspecific IV conduction delay	46 (6.6%)	25 (11.6%)	16 (13.8%)	
Low voltages	156 (21.2%)	63 (28.3%)	70 (18.8%)	0.007
Signs of LVH	68 (9.3%)	31 (14.1%)	24 (6.5%)	0.002
Echocardiography				
LVEF (%)	52.1 ± 12.2	39.5 ± 7.9	60.0 ± 6.5	<0.001
IVSd (mm)	15.6 ± 4.3	17.1 ± 3.6	15.4 ± 4.2	<0.001
PWT (mm)	14.1 ± 4.0	15.3 ± 3.5	14.1 ± 4.0	<0.001
LAVI (mL/m^2^)	49.3 ± 24.3	55.9 ± 29.1	46.6 ± 20.0	<0.001
Indexed stroke volume (mL/m^2^)	31.6 ± 11.0	26.8 ± 8.8	34.9 ± 11.2	<0.001
LV GLS (%)	−13.3 ± 4.7	−9.7 ± 2.9	−14.6 ± 4.2	<0.001
RELAS	2.0 ± 0.9	2.0 ± 1.0	2.1 ± 0.8	0.898
TAPSE	18.2 ± 5.2	15.6 ± 4.3	19.6 ± 10.9	<0.001
RA area (cm^2^)	20.8 ± 6.6	23.0 ± 6.6	19.7 ± 6.2	<0.001
E/e’	17.5 ± 10.2	19.8 ± 10.9	16.5 ± 9.6	<0.001
Medication				
Beta-blocker	346 (43.7%)	139 (56.0%)	161 (40.8%)	<0.001
ACEi/ARB	326 (41.2%)	109 (44.0%)	157 (39.7%)	0.292
Diuretics	418 (52.8%)	180 (72.9%)	175 (44.2%)	<0.001
MRA	204 (25.8%)	88 (35.5%)	87 (22.0%)	<0.001
Disease-modifying treatment	315 (44.9%)	95 (43.4%)	169 (47.6%)	0.324

ACEi/ARB: angiotensin-converting enzyme inhibitor/angiotensin receptor blocker; AF: atrial fibrillation; ATTR-CA: transthyretin cardiac amyloidosis; CAD: coronary artery disease; CKD: chronic kidney disease; DM: diabetes mellitus; E/e’: mitral inflow E-wave divided by annular tissue e’ wave; eGFR: estimated glomerular filtration rate; HS: high-sensitivity; IV; intraventricular; IVSd: interventricular septum thickness in diastole; LAVI: left atrial volume index; LBBB: left bundle branch block; LV GLS: left ventricle global longitudinal strain; LVEF: left ventricle ejection fraction; LVH: left ventricular hypertrophy; MRA: mineralocorticoid receptor antagonist; NAC: National Amyloidosis Centre; NT-proBNP: N-Terminal pro-B-type natriuretic peptide; NYHA: New York Heart Association; PWT: posterior wall thickness; RA: right atrial; RBBB: right bundle branch block; RELAS: relative apical sparing ratio; TAPSE: tricuspid annular plane systolic excursion; TIA: transient ischemic attack.

**Table 2:** Baseline and echocardiographic characteristics of patients treated and not treated with beta-blockers and ACEi/ARBs.

	Patients Treated with Beta-Blockers (*n* = 346, 43.7%)	Patients Not Treated with Beta-Blockers (*n* = 445, 56.3%)	*p*-Value	Patients Treated with ACEi/ARBs (*n* = 326, 41.2%)	Patients Not Treated with ACEi/ARBs (*n* = 465, 58.8%)	*p*-Value
Sex (male)	262 (75.5%)	316 (71.0%)	0.138	254 (77.9%)	324 (69.7%)	0.010
Age	81 (76–86)	80 (72–84)	<0.001	81 (77–84)	80 (72–85)	<0.001
AF/flutter	179 (60.3%)	149 (36.8%)	<0.001	157 (54.0%)	171 (41.6%)	0.001
CAD	98 (28.3%)	80 (18.0%)	<0.001	90 (27.6%)	88 (18.9%)	0.004
DM	66 (19.2%)	55 (12.4%)	<0.001	58 (17.8%)	63 (13.6%)	0.104
Hypertension	212 (61.4%)	203 (45.8%)	<0.001	233 (71.7%)	182 (39.3%)	<0.001
Stroke/TIA	47 (17.7%)	40 (10.4%)	0.008	43 (16.6%)	44 (11.3%)	0.051
NYHA class			<0.001			0.108
I	69 (21.4%)	148 (38.5%)		81 (26.7%)	136 (33.7%)	
II	170 (52.8%)	163 (42.4%)		147 (48.5%)	186 (46.2%)	
III	73 (22.7%)	65 (16.9%)		64 (21.1%)	74 (18.4%)	
IV	10 (3.1%)	8 (2.1%)		11 (3.1%)	7 (1.7%)	
NAC stage			<0.001			0.048
I	109 (48.0%)	207 (67.9)		116 (53.5%)	200 (63.5%)	
II	54 (23.8%)	70 (23.0%)		61 (28.1%)	63 (20.0%)	
III	64 (28.2%)	28 (9.2%)		40 (18.4%)	52 (16.5%)	
NT-proBNP (ng/L)	3195 (1207–6932)	1882 (768–3892)	<0.001	2796 (1074–5917)	2285 (901–5106)	<0.001
HS Troponin (ng/L)	49 (31–92)	42 (22–67)	<0.001	49 (34–88)	46 (24–82)	0.062
eGFR (mL/min/1.73 m^2^)	57.8 ± 22.6	71.5 ± 25.3	<0.001	60.8 ± 22.1	68.6 ± 26.6	<0.001
CKD stage 3–5	182 (55.8%)	235 (33.1%)	<0.001	153 (50.7%)	164 (38.0%)	<0.001
Systolic blood pressure (mmHg)	128.9 ± 23.1	126.3 ± 19.9	0.139	129.2 ± 21.7	126.3 ± 21.2	0.095
Diastolic blood pressure (mmHg)	73.9 ± 14.1	73.8 ± 16.9	0.819	74.3 ± 12.3	73.4 ± 13.8	0.399
Electrocardiographic parameters						
Rhythm at baseline			0.097			0.116
Sinus rhythm	189 (55.6%)	278 (63.0%)		183 (56.7%)	284 (62.0%)	
AF/flutter	124 (36.5%)	124 (28.1%)		114 (35.3%)	134 (29.3%)	
Paced rhythm	25 (7.4%)	37 (8.4%)		26 (8.0%)	36 (7.9%)	
Heart rate (bpm)	73.8 ± 16.9	75.4 ± 15.0	0.152	73.5 ± 15.3	75.6 ± 16.2	0.077
Conduction abnormalities			0.012			0.565
LBBB	55 (18.4%)	69 (17.5%)		52 (18.7%)	72 (17.3%)	
RBBB	53 (17.7%)	41 (10.4%)		42 (15.1%)	52 (12.5%)	
Aspecific IV conduction delay	23 (7.7%)	22 (5.6%)		20 (7.2%)	25 (6.0%)	
Low voltages	75 (23.1%)	81 (19.9%)	0.280	65 (21.6%)	91 (21.1%)	0.876
Signs of LVH	28 (8.7%)	40 (9.9%)	0.585	31 (10.3%)	37 (8.6%)	0.435
Echocardiographic parameters						
LVEF (%)	50.0 ± 11.8	53.8 ± 12.2	<0.001	51.2 ± 11.8	52.2 ± 12.4	0.164
IVSd (mm)	16.5 ± 3.8	15.0 ± 4.6	<0.001	16.4 ± 3.8	15.1 ± 4.5	<0.001
PWT (mm)	14.8 ± 4.0	13.6 ± 3.9	<0.001	14.6 ± 4.1	13.8 ±3.9	0.008
LAVI (mL/m^2^)	53.3 ± 27.9	46.2 ± 20.6	0.001	52.5 ± 28.3	46.9 ± 20.6	0.011
Indexed stroke volume (mL/m^2^)	30.7 ± 11.4	32.3 ± 10.4	0.123	31.9 ± 11.4	31.2 ± 10.5	0.541
LV GLS (%)	−12.2 ± 4.5	−14.0 ± 4.7	<0.001	−12.4 ± 4.2	−13.8 ± 4.9	<0.001
RELAS	2.03 ± 0.97	2.02 ± 0.79	0.900	2.09 ± 0.94	1.98 ± 0.82	0.191
TAPSE	17.5 ± 5.0	18.8 ± 5.3	0.003	18.1 ± 5.1	18.3 ± 5.3	0.554
RA area (cm^2^)	21.7 ± 7.1	20.1 ± 6.1	0.008	21.7 ± 6.4	20.1 ± 6.6	0.010
E/e’	18.6 ± 5.3	16.6 ± 9.0	0.022	17.9 ± 10.2	17.2 ± 10.3	0.448
Medication						
Diuretics	234 (68.0%)	183 (41.1%)	<0.001	218 (67.3%)	199 (42.8%)	<0.001
MRA	108 (31.2%)	96 (21.6%)	0.002	102 (31.3%)	102 (21.9%)	0.003
Disease-modifying treatment	115 (38.3%)	199 (49.9%)	0.002	117 (41.6%)	197 (47.1%)	0.152

ACEi/ARB: angiotensin-converting enzyme inhibitor/angiotensin receptor blocker; AF: atrial fibrillation; CAD: coronary artery disease; CKD: chronic kidney disease; DM: diabetes mellitus; E/e’: mitral inflow E-wave divided by annular tissue e’ wave; eGFR: estimated glomerular filtration rate; HF: heart failure; HS: high-sensitivity; IV: intraventricular; IVSd: interventricular septum thickness in diastole; LAVI: left atrial volume indexed; LV GLS: left ventricle global longitudinal strain; LVEF: left ventricle ejection fraction; LVH: left ventricular hypertrophy; MRA: mineralocorticoid receptor antagonist; NAC: National Amyloidosis Centre; NT-proBNP: N-Terminal pro-B-type natriuretic peptide; NYHA: New York Heart Association; PWT: posterior wall thickness; RA: right atrial; RELAS: relative apical sparing ratio; TAPSE: tricuspid annular plane systolic excursion; TIA: transient ischemic attack.

**Table 3:** Combination heart failure therapy, split by period of diagnosis.

	Overall Population (*n* = 791)	Before 2016 (*n* = 136)	2016–2021 (*n* = 493)	After 2021 (*n* = 107)	*p*-Value
BB and ACE	115 (14.5%)	11 (8.1%)	84 (17.0%)	19 (17.8%)	0.030
BB and MRA	40 (5.1%)	5 (3.7%)	28 (5.7%)	7 (6.5%)	0.568
ACEi/ARB and MRA	34 (4.3%)	5 (3.7%)	19 (3.9%)	10 (9.3%)	0.042
BB, ACEi/ARB, and MRA	68 (8.6%)	11 (8.1%)	45 (9.1%)	12 (11.2%)	0.698
No use of BB, ACEi/ARB, or MRA	240 (30.3%)	61 (44.9%)	117 (23.7%)	20 (18.7%)	<0.001

ACEi/ARB: angiotensin-converting enzyme inhibitor/angiotensin receptor blocker; BB: beta-blocker; MRA: mineralocorticoid receptor antagonist.

**Table 4:** Hazard ratios in multivariate Cox regression with included covariables (age, AF, CAD, DM, wild-type phenotype (vs. variant phenotype), NAC stage, LV GLS, beta-blocker, and ACEi/ARB).

	Beta-Blocker		ACEi/ARB	
	HR (95% CI)	*p*-Value	HR (95% CI)	*p*-Value
Age at diagnosis	1.073 (1.028–1.120)	<0.001	1.074 (1.028–1.121)	0.001
AF	1.977 (0.884–4.419)	0.097	1.887 (0.851–4.184)	0.118
CAD	1.131 (0.592–2.163)	0.709	1.102 (0.580–2.096)	0.767
DM	0.528 (0.203–1.374)	0.191	0.526 (0.201–1.375)	0.190
Wild-type	0.678 (0.678–0.193)	0.544	0.669 (0.193–2.321)	0.669
NAC stage				
I vs. II	1.009 (0.395–2.580)	0.985	1.115 (0.447–2.782)	0.816
I vs. III	4.702 (2.022–910.937)	<0.001	4.302 (1.873–9.882)	<0.001
LV GLS	00.928 (0.851–1.013)	0.095	0.933 (0.857–1.016)	0.111
Beta-blocker	0.666 (0.352–1.262)	0.213	-	-
ACEi/ARB	-	-	0.878 (0.490–1.575)	0.663

ACEi/ARB: angiotensin-converting enzyme inhibitor/angiotensin receptor blocker; AF: atrial fibrillation; CAD: coronary artery disease; DM: diabetes mellitus; HR: hazard ratio; LV GLS: left ventricle global longitudinal strain; NAC: National Amyloidosis Centre.

## Data Availability

The data that support the results of this study are available from the corresponding author upon reasonable request.

## Supplementary Materials

The following supporting information can be downloaded at: https://www.mdpi.com/article/10.3390/jcdd12100403/s1, Table S1: Univariate testing of variables for Cox proportional hazard survival analysis; Figure S1: Kaplan-Meier curves of beta-blocker (**a**,**b**) and ACEi/ARB (**c**,**d**) prescription in ATTR-CA patients, split by wild-type and variant phenotype: (**a**) Survival in patients treated with beta-blockers in wild-type ATTR-CA patients; (**b**) Survival in patients treated with beta-blockers in variant ATTR-CA patients; (**c**) Survival in patients treated with ACEi/ARBs in wild-type ATTR-CA patients; (**d**) Survival in patients treated with ACEi/ARBs in variant ATTR-CA patients. ACEi/ARB: angiotensin-converting enzyme inhibitor/ angiotensin receptor blocker; ATTR-CA: transthyretin cardiac amyloidosis; BB: beta-blocker.

## References

[1] Gentile L., Coelho T., Dispenzieri A., Conceição I., Waddington-Cruz M., Kristen A., Wixner J., Diemberger I., Gonzalez-Moreno J., Cariou E. (2023). A 15-year consolidated overview of data in over 6000 patients from the Transthyretin Amyloidosis Outcomes Survey (THAOS). Orphanet J. Rare Dis..

[2] Ioannou A., Patel R.K., Razvi Y., Porcari A., Sinagra G., Venneri L., Bandera F., Masi A., Williams G.E., O’bEara S. (2022). Impact of Earlier Diagnosis in Cardiac ATTR Amyloidosis Over the Course of 20 Years. Circulation.

[3] Teresi L., Trimarchi G., Liotta P., Restelli D., Licordari R., Carciotto G., Francesco C., Crea P., Dattilo G., Micari A. (2024). Electrocardiographic Patterns and Arrhythmias in Cardiac Amyloidosis: From Diagnosis to Therapeutic Management-A Narrative Review. J. Clin. Med..

[4] Maurer M.S., Schwartz J.H., Gundapaneni B., Elliott P.M., Merlini G., Waddington-Cruz M., Kristen A.V., Grogan M., Witteles R., Damy T. (2018). Tafamidis Treatment for Patients with Transthyretin Amyloid Cardiomyopathy. N. Engl. J. Med..

[5] Gillmore J.D., Judge D.P., Cappelli F., Fontana M., Garcia-Pavia P., Gibbs S., Grogan M., Hanna M., Hoffman J., Masri A. (2024). Efficacy and Safety of Acoramidis in Transthyretin Amyloid Cardiomyopathy. N. Engl. J. Med..

[6] Fontana M., Berk J.L., Gillmore J.D., Witteles R.M., Grogan M., Drachman B., Damy T., Garcia-Pavia P., Taubel J., Solomon S.D. (2025). Vutrisiran in Patients with Transthyretin Amyloidosis with Cardiomyopathy. N. Engl. J. Med..

[7] Ponikowski P., Voors A.A., Anker S.D., Bueno H., Cleland J.G.F., Coats A.J.S., Falk V., González-Juanatey J.R., Harjola V.P., Jankowska E.A. (2016). 2016 ESC Guidelines for the diagnosis and treatment of acute and chronic heart failure: The Task Force for the diagnosis and treatment of acute and chronic heart failure of the European Society of Cardiology (ESC)Developed with the special contribution of the Heart Failure Association (HFA) of the ESC. Eur. Heart J..

[8] McDonagh T.A., Metra M., Adamo M., Gardner R.S., Baumbach A., Böhm M., Burri H., Butler J., Čelutkienė J., Chioncel O. (2021). 2021 ESC Guidelines for the diagnosis and treatment of acute and chronic heart failure. Eur. Heart J..

[9] Garcia-Pavia P., Rapezzi C., Adler Y., Arad M., Basso C., Brucato A., Burazor I., Caforio A.L.P., Damy T., Eriksson U. (2021). Diagnosis and treatment of cardiac amyloidosis: A position statement of the ESC Working Group on Myocardial and Pericardial Diseases. Eur. Heart J..

[10] Kittleson M.M., Ruberg F.L., Ambardekar A.V., Brannagan T.H., Cheng R.K., Clarke J.O., Dember L.M., Frantz J.G., Hershberger R.E., Maurer M.S. (2023). 2023 ACC Expert Consensus Decision Pathway on Comprehensive Multidisciplinary Care for the Patient With Cardiac Amyloidosis: A Report of the American College of Cardiology Solution Set Oversight Committee. J. Am. Coll. Cardiol..

[11] Ioannou A., Massa P., Patel R.K., Razvi Y., Porcari A., Rauf M.U., Jiang A., Cabras G., Filisetti S., Bolhuis R. (2023). Conventional heart failure therapy in cardiac ATTR amyloidosis. Eur. Heart J..

[12] Gillmore J.D., Damy T., FontAna M., Hutchinson M., Lachmann H.J., Martinez-Naharro A., Quarta C.C., Rezk T., Whelan C.J., Gonzalez-Lopez E. (2018). A new staging system for cardiac transthyretin amyloidosis. Eur. Heart J..

[13] Phelan D., Collier P., Thavendiranathan P., Popović Z.B., Hanna M., Plana J.C., Marwick T.H., Thomas J.D. (2012). Relative apical sparing of longitudinal strain using two-dimensional speckle-tracking echocardiography is both sensitive and specific for the diagnosis of cardiac amyloidosis. Heart.

[14] Malgie J., Wilde M.I., Clephas P.R., Emans M.E., Koudstaal S., Schaap J., Mosterd A., van Ramshorst J., Wardeh A.J., van Wijk S. (2024). Contemporary guideline-directed medical therapy in de novo, chronic, and worsening heart failure patients: First data from the TITRATE-HF study. Eur. J. Heart Fail..

[15] Ramsell S., Bermudez C.A., Baiyee C.A.M.T., Rodgers B., Parikh S., Almaani S., Sharma N., LoRusso S., Freimer M., Redder E. (2022). Beta-Adrenergic Antagonist Tolerance in Amyloid Cardiomyopathy. Front. Cardiovasc. Med..

[16] Negreira-Caamano M., Río J.M.-D., Morón-Alguacil A., Pérez-Díaz P., Piqueras-Flores J. (2023). Starting sacubitril-valsartan is safe in patients with transthyretin cardiac amyloidosis and impaired ejection fraction. Rev. Port. Cardiol..

[17] Foa A., Vaduganathan M., Claggett B.L., Pabon M.A., Lu H., Pfeffer M.A., Packer M., Vardeny O., Rouleau J.L., Lefkowitz M. (2024). Sacubitril/Valsartan-Related Hypotension in Patients With Heart Failure and Preserved or Mildly Reduced Ejection Fraction. J. Am. Coll. Cardiol..

[18] Porcari A., Cappelli F., Nitsche C., Tomasoni D., Sinigiani G., Longhi S., Bordignon L., Masri A., Serenelli M., Urey M. (2024). SGLT2 Inhibitor Therapy in Patients With Transthyretin Amyloid Cardiomyopathy. J. Am. Coll. Cardiol..

[19] Sivamurugan A., Byer S.H., Grewal U.S., Dominic P. (2024). Impact of SGLT2 inhibitors on the outcomes of patients with cardiac arrhythmias and transthyretin cardiac amyloidosis. Heart Rhythm..

[20] Cheng R.K., Vasbinder A., Levy W.C., Goyal P., Griffin J.M., Leedy D.J., Maurer M.S. (2021). Lack of Association Between Neurohormonal Blockade and Survival in Transthyretin Cardiac Amyloidosis. J. Am. Heart Assoc..

[21] Barge-Caballero G., Barge-Caballero E., López-Pérez M., Bilbao-Quesada R., González-Babarro E., Gómez-Otero I., López-López A., Gutiérrez-Feijoo M., Varela-Román A., González-Juanatey C. (2022). Beta-Blocker Exposure and Survival in Patients With Transthyretin Amyloid Cardiomyopathy. Mayo Clin. Proc..

[22] Kwok C.S., Choy C.H., Pinney J., Townend J.N., Whelan C., Fontana M., Gillmore J.D., Steeds R.P., Moody W.E. (2024). Effect of beta-blockade on mortality in patients with cardiac amyloidosis: A systematic review and meta-analysis. ESC Heart Fail..

[23] Aimo A., Vergaro G., Castiglione V., Rapezzi C., Emdin M. (2020). Safety and Tolerability of Neurohormonal Antagonism in Cardiac Amyloidosis. Eur. J. Intern. Med..

