# Supplementary material for: Trends in Conventional Heart Failure Therapy in a Real-World Multinational ATTR-CA Cohort
Source: J Cardiovasc Dev Dis. 2025 Oct 11;12(10):403. doi: 10.3390/jcdd12100403 (PMC12565287; doi:10.3390/jcdd12100403)
Supplement: Supplementary file 1 [file jcdd-12-00403-s001.zip › jcdd-3904518-supplementary-proof/jcdd-3904518-supplementary-proof.pdf]

Supplementary materials

Table S1. Univariate testing of variables for Cox proportional hazard survival analysis.

	HR [95% CI]	p-value
Age at diagnosis	1.078 [1.060 – 1.096]	<0.001
AF	2.618 [1.859 – 3.685]	<0.001
CAD	1.894 [1.372 – 2.615]	<0.001
DM	1.639 [1.139-2.359]	0.008
NAC stage		
I vs. II	3.910 [2.298 – 6.653]	<0.001
I vs. III	10.250 [6.077 – 17.289]	<0.001
LV GLS	0.865 [0.827-0.905]	<0.001
Wild-type phenotype	7.594 [4.210 – 13.697]	<0.001
Beta-blocker	1.445 [1.068-1.957]	0.017
ACEi/ARB	1.105 [0.816-1.495]	0.519

ACEi/ARB: angiotensin-converting enzyme inhibitor/angiotensin receptor blocker; AF: atrial fibrillation; CAD: coronary artery disease; DM: diabetes mellitus; HR: hazard ratio; LV GLS: left ventricle global longitudinal strain; NAC: National Amyloidosis Centre.

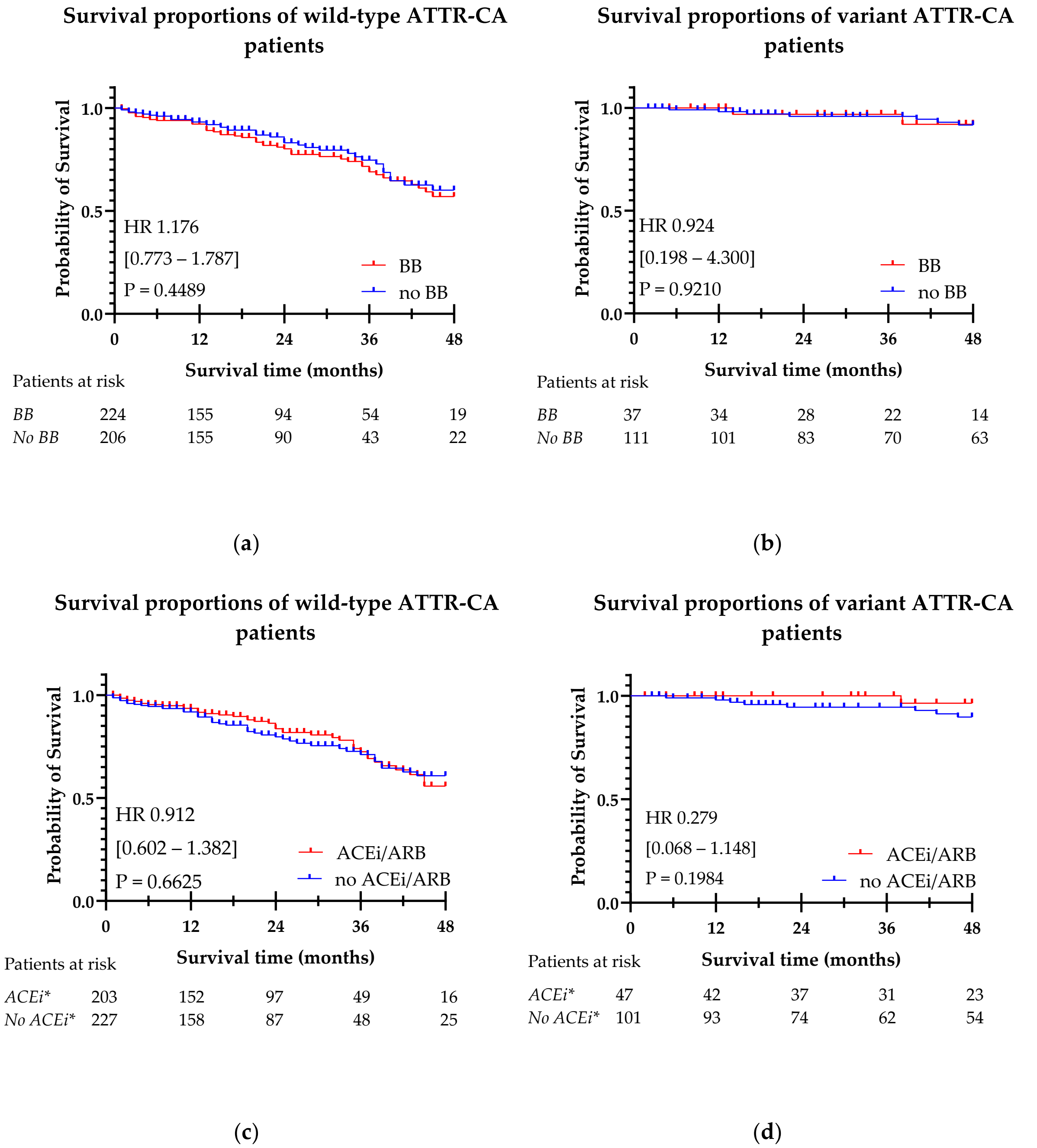

Figure S1. Kaplan-Meier curves of beta-blocker (a, b) and ACEi/ARB (c, d) prescription in ATTR-CA patients, split by wild-type and variant phenotype: (**a**) Survival in patients treated with beta-blockers in wild-type ATTR-CA patients; (**b**) Survival in patients treated with beta-blockers in variant ATTR-CA patients; (c) Survival in patients treated with ACEi/ARBs in wild-type ATTR-CA patients; (**d**) Survival in patients treated with ACEi/ARBs in variant ATTR-CA patients. ACEi/ARB: angiotensin-converting enzyme inhibitor/ angiotensin receptor blocker; ATTR-CA: transthyretin cardiac amyloidosis; BB: beta-blocker. *ACEi/ARB
